# Editorial: Clinical pharmacist service promotes the improvement of medical quality

**DOI:** 10.3389/fphar.2024.1418181

**Published:** 2024-04-26

**Authors:** Hao Li, Wei Luan, Zhi-Chun Gu, Shusen Sun, Jianrong Zhang

**Affiliations:** ^1^ Clinical Research Ward, Clinical Research Center, Shanghai Children’s Medical Center, School of Medicine, Shanghai, China; ^2^ Department of Nursing, Shuguang Hospital, Shanghai University of Traditional Chinese Medicine, Shanghai, China; ^3^ Department of Pharmacy, Ren Ji Hospital, Shanghai Jiao Tong University School of Medicine, Shanghai, China; ^4^ Department of Pharmacy Practice, College of Pharmacy and Health Sciences, Western New England University, Springfield, MA, United States; ^5^ Melbourne Medical School, Faculty of Medicine, Dentistry and Health Sciences, University of Melbourne, Melbourne, Australia

**Keywords:** clinical pharmacist, medication therapy management, pharmaceutical care, pharmacovigilance, medical education and counseling

## Introduction

Clinical pharmacists play a vital role in the healthcare system, improving the rational use of medications (Gu et al., 2018) then clinical outcomes (Shi et al., 2021; Shi et al., 2023), and fostering unity within the multidisciplinary teams (Shi et al., 2020). This Research Topic “Clinical Pharmacist Service Promotes the Improvement of Medical Quality” highlights the role of clinical pharmacists, featuring 26 articles (23 original research articles, one brief research report, one policy and practice review, and one systematic review). These articles explore various aspects of clinical pharmacy services, including providing medication therapy management (MTM) services and pharmaceutical care, expanding pharmacovigilance information, participating in clinical trials, and filling gaps in knowledge regarding medical education and counselling.

## Medication Therapy Management Service and Pharmaceutical Care

A systematic evaluation of the role of clinical pharmacists provides a deeper understanding of their value. Deng et al. conducted a systematic review and meta-analysis to assess the multifaceted benefits of MTM services, considering economic, clinical, and humanistic outcomes. Their findings suggest that MTM services provided by pharmacists significantly improve clinical outcomes, notably by reducing readmission rates, emergency department visits, adverse drug events, and the length of hospital stays. However, these services are less effective in achieving economic and humanistic outcomes.

Several high-quality clinical studies have been conducted to confirm the role of clinical pharmacists in disease management. Khan et al. carried out a randomized, usual care-controlled study in Pakistan to evaluate the impact of pharmacist-led interventions on health-related quality of life (HRQOL) among tuberculosis patients using the EuroQol-5D-3L instrument. They found that patient-centered care interventions led by pharmacists significantly improved (HRQOL), supporting the inclusion of clinical pharmacists in interdisciplinary teams for tuberculosis management. In Japan, Suzuki et al. demonstrated through a multicenter prospective observational study that hospital pharmacist interventions effectively promoted appropriate prescription practices, reducing the number of medications used, and decreasing polypharmacy. Cheng et al. Evaluated the role of clinical pharmacists in anti-infective treatment for patients with central nervous system infections through a single-center retrospective cohort study, finding improvements in the effectiveness and appropriateness of treatment. Yu et al. assessed the impact of a clinical pharmacist-led antimicrobial stewardship program in a neurosurgical intensive care unit through a cohort study, noting a significant reduction in critical antibacterial agents and multi-drug resistant organism infections. Lastly, Gao et al. explored the effects of continuous pharmaceutical care led by clinical pharmacists on medication adherence and clinical outcomes in coronary heart disease patients through a prospective cohort study. Their findings indicated significantly higher medication adherence and improved clinical indicators, such as low-density lipoprotein cholesterol and blood pressure, with a substantially lower incidence of adverse drug reactions. Although there were no statistical differences in overall readmission rates, the intervention group showed a trend toward lower rates.

Establishing and verifying effective pharmaceutical care models can significantly improve clinical treatment. Xie et al. developed a model for managing cancer pain in primary care settings in China using the Delphi method. This model was shown to significantly increase the rate and accuracy of pain assessments, reduce pain scores, and decrease the incidence of adverse reactions. In Australia, Atey et al. piloted a partnered pharmacist medication charting system in the emergency department of a tertiary hospital, which significantly reduced potentially inappropriate medication use among older patients in the emergency department. However, this benefit was not observed at hospital discharge, suggesting the need for greater interprofessional collaborations. The same model was also associated with significantly lower relative stay indices and admission costs, saving an average of $1,269 per admission. Despite these economic benefits, the model did not affect clinical outcomes.

## Pharmacovigilance study

New pharmacovigilance information can help clinical pharmacists better anticipate drug risks and control adverse drug events. Deng et al. used the US Food and Drug Administration Adverse Event Reporting System (FAERS) to quantify the risk of rhabdomyolysis associated with newer-generation anti-seizure medications, identifying seven medications related to this condition. Similarly, He et al. investigated diazepam-related adverse events using FAERS data, uncovering significant unlisted effects such as congenital nystagmus, developmental delays, and rhabdomyolysis. Gao et al. analyzed the clinical characteristics and risk factors for aripiprazole-induced liver injury using the China Adverse Drug Reaction Monitoring System. Furthermore, Jiang et al. developed a pharmacist-led risk management service to predict and manage risks associated with infusions of iodinated contrast media. This system, operated by a multidisciplinary team, provides personalized risk identification and assessment before infusion, effectively minimizing adverse reactions.

## Participation in clinical trials

Clinical pharmacists play an integral role in clinical trials by formulating trial plans and overseeing pharmaceutical aspects to ensure drug safety. Lu et al. assessed the minimum plasma concentration and safety of nirmatrelvir/ritonavir in patients with hemodialysis and mild COVID-19. They discovered that the two dose regimens of nirmatrelvir/ritonavir were excessive for such patients, suggesting a need to optimize the medication regimen for this group. Based on this research, Yan et al. investigated the effectiveness and safety of nirmatrelvir/ritonavir in patients with kidney dysfunction and COVID-19. This trial included consultations with pharmacists about prescription safety and dose adjustments. Furthermore, Hu et al. evaluated the impact of body mass index on responses to ABVD-like chemotherapy in newly diagnosed patients with Hodgkin lymphoma. Their findings indicate that the body mass index is an independent risk factor for a poorer overall response, highlighting the need for personalized cancer treatment plans for obese or overweight patients.

## Medical education and counseling

Detecting gaps in medication knowledge is essential to improve medical education and counseling. Chang et al. translated and validated the Jessa Atrial Fibrillation Knowledge Questionnaire (JAKQ) and assessed patients’ knowledge of atrial fibrillation and oral anticoagulation. They found that the Chinese version of JAKQ was reliable and valid, making it a valuable tool for assessing patient knowledge. In contrast, Xie et al. reported that nurses in China had a limited understanding of inhalation therapy and the use of inhaler devices, suggesting the need for pharmacists to strengthen training in these areas, especially in hospitals without clinical pharmacists. Alorfi et al. assessed the knowledge of drug-drug interactions among community pharmacists in Jeddah, Saudi Arabia, using a self-administered questionnaire and discovered that most could not correctly answer questions on this topic, underscoring the importance of ongoing training and education. Lu et al. used a questionnaire to explore attitudes, involvement, and knowledge of clinical pharmacists in Guangdong province, China, about postoperative acute pain services. They found a positive attitude but insufficient participation, indicating the need for systematic training and established work protocols. Rendrayani et al. evaluated knowledge, attitudes, and practices regarding medication therapy management among pharmacists at community health centers in Indonesia, revealing high levels of expertise but inadequate attitudes and practices, suggesting that direct participation or training programs are necessary to improve skills and collaboration with healthcare professionals. Lastly, Zheng et al. conducted a cross-sectional study on the appropriateness of anticoagulation therapy among patients with nonvalvular atrial fibrillation, identifying issues with underdosing, particularly in patients with renal dysfunction or paroxysmal atrial fibrillation.

Identifying barriers in pharmaceutical care on time is crucial to improving clinical pharmacist training programs. Chen et al. developed and validated an instrument to measure pharmaceutical care barriers in Chinese hospitals, providing administrators and policymakers with a reliable tool to guide improvements in the pharmaceutical care system. Robberechts et al. created a quality assessment instrument for type 3 medication reviews, consisting of eight domains, using an electronic questionnaire distributed to pharmacists, pharmacy academics, pharmacists trained in medication review, and pharmacy students. They highlighted eight critical statements as essential, such as employing understandable language in patient conversations. Jairoun et al. assessed the pharmaceutical care status of community pharmacists for cardiovascular disease patients in the United Arab Emirates using a structured questionnaire in a cross-sectional study. They found that community pharmacists actively contribute to managing and preventing cardiovascular diseases. They recommend that pharmacists in the United Arab Emirates improve their participation in other healthcare services, such as monitoring treatment responses, reviewing medication refill histories, and providing medication counselling.

The application of information technology tools can enhance medication guidance. Liu et al. developed a WeChat-based Internet pharmacy service platform named Xiang Medicine Guidance, designed to offer medication guidance, reminders, and consultation services. They discovered that this platform significantly improved medication adherence and allowed patients to better manage their medications.

## Future perspectives

As expected, we have enjoyed widespread participation from clinical pharmacists worldwide in this Research Topic. We acknowledge that many new perspectives and practices are urgently needed to advance the field of clinical pharmacy. This initiative aims to facilitate improved communication and learning among clinical pharmacists globally. We eagerly anticipate further engagement with our international colleagues in the second season of this particular Research Topic in the future.
